# The Effect of Diclofenac on Bleeding, Platelet Function, and Consumption of Opioids Following Cardiac Surgery

**DOI:** 10.21470/1678-9741-2019-0283

**Published:** 2020

**Authors:** Irena Osojnik, Mirt Kamenik

**Affiliations:** 1 Department of Anaesthesiology, Intensive Care and Pain Management, University Medical Centre Maribor, Maribor, Slovenia.; 2 Department of Anesthesia and Reanimation, Faculty of Medicine, University of Maribor, Maribor, Slovenia.

**Keywords:** Coronary Surgical Procedures, Cardiac Artery Bypass, C-Reactive Protein, Blood Platelets, Platelet Function Tests, Magnesium, Radioisotopes

## Abstract

**Objective:**

To establish whether the use of diclofenac reduces the administration of opioids and how it affects bleeding and platelet function after the coronary artery bypass grafting (CABG) surgery with use of cardiopulmonary bypass (CPB).

**Methods:**

A total of 72 patients undergoing CABG surgery were included in this retrospective randomized study and divided into two groups (34 patients received diclofenac and the control group of 38 patients did not). For postoperative analgesia, both groups were prescribed opioids (piritramide). The primary endpoint was to establish the consumption of opioids. The secondary endpoint was to determine bleeding and the function of platelets 20 hours after the surgery.

**Results:**

The consumption of piritramide (diclofenac group 26±8 mg *vs*. control group 28±8 mg), the blood loss, and the function of platelets did not significantly differ between the groups within 20 hours after surgery. C-reactive protein (CRP) was statistically significantly lower in the diclofenac group than in the control group (33±15 mg/L *vs*. 46±22 mg/L, respectively, *P*<0.05).

**Conclusion:**

The study concluded that patients administered with diclofenac after the heart surgery did not consume less opioid analgesics and did not exhibit less symptoms linked to the consumption of opioids. Diclofenac in clinically administered doses does not interfere with the function of platelets and does not cause increased bleeding. Lower CRP in the diclofenac group may indicate a reduced inflammatory response after CPB. Therefore, diclofenac could be safe for use in patients undergoing CABG surgery but its value in reducing opioid consumption should be questioned.

**Table:** 

Abbreviations, acronyms & symbols				
AF	= Atrial fibrillation		i.v.	= Intravenous
ANOVA	= Analysis of variance		NCA	= Nurse-controlled analgesia
aPTT	= Activated partial thromboplastin time		NRS	= Numerical rating scale
AVR	= Aortic valve replacement		NSAIDs	= Nonsteroidal anti-inflammatory drugs
CABG	= Coronary artery bypass grafting		NYHA	= New York Heart Association
CONSORT	= Consolidated Standards of Reporting Trials		PCA	= Patient-controlled analgesia
COPD	= Chronic obstructive pulmonary disease		PP	= Platelet plasma
CPB	= Cardiopulmonary bypass		PT	= Prothrombin time
CRP	= C-reactive protein		RBC	= Red blood cells
EF	= Ejection fraction		S	= Sufentanil
EuroSCORE II or ES II	= European System for Cardiac Operative Risk Evaluation		SD	= Standard deviation
F	= Fentanyl		SR	= Sufentanil and Remifentanil
FFP	= Fresh frozen plasma		TT	= Thrombin time
ICU	= Intensive care unit		VAS	= Visual analogue scale

## INTRODUCTION

Ischemic heart disease often requires treatment by the coronary artery bypass grafting (CABG) surgery using cardiopulmonary bypass (CPB). The anesthesiologist uses an anesthesiologic technique during surgery to ensure sufficient postoperative analgesia, which is continued at the intensive care unit (ICU)^[1]^. Pain after cardiac surgery is associated with sternotomy, pericardiotomy, insertion of thoracic drains, and the removal of a vein from a patient's leg^[2,3]^. It may be due to inflammation in the thoracic cavity and inflammation of the parietal pleura or the consequence of postoperative pericarditis.

Sufficient postoperative analgesia prevents the patient's discomfort, reduces morbidity, reduces the length of hospitalization, and thus reduces the cost of treatment. Insufficient analgesia results in a stress response that has adverse effects on important organ systems, such as the central nervous system, circulatory system, metabolism, and hemostasis in the patient after surgery^[4]^. A modern treating method of pain is multimodal analgesia, which means the use of active substances and techniques that work through different mechanisms and thus have less side effects and greater analgesic efficacy than a single drug^[5]^. The choice of an individual drug, its dose, the route of administration, and the duration of treatment are always adapted to each patient. One of the methods in the multimodal approach to pain treatment after a heart surgery is to add nonsteroidal anti-inflammatory drugs (NSAIDs) to opioid analgesics^[6]^. The use of NSAIDs results in decreased consumption of opioid analgesics and their potential side effects after the surgery; however, their usage can contribute to increased bleeding, impaired kidney function, and possible ischemic events^[7]^.

One of the common complications following a heart surgery using CPB is bleeding^[8]^. The cause of bleeding can be surgical and/ or non-surgical. The surgical cause is the result of unsatisfactory surgical hemostasis. The non-surgical cause of bleeding is due to the effects of CPB on blood clotting or the action of the drugs that the patient received before surgery (heparin, clopidogrel, aspirin, platelet receptor antagonists, NSAIDs, etc.). Qualitative platelet disorders are also occurring during CPB. The surfaces of the system of extracorporeal circulation, heparin, and hypothermia cause activation and secretion of platelets. The level of dysfunction of the platelet function coincides with the duration of CPB and the degree of hypothermia.

Diclofenac is a chemical derivative of carboxylic acid and has, in tissue damage, analgesic, antipyretic, and anti-inflammatory effects by inhibiting the isoform of the enzymes COX-1 and COX-2. Diclofenac also influences the platelet function^[9]^, thereby potentially increasing the risk of bleeding after surgery.

The purpose of our study was to evaluate whether the use of diclofenac reduces the use of opioids, reduces the side effects of opioids, and shortens the time until the respiratory tube is removed. In the study, we also wanted to determine to what extent the administration of diclofenac interferes with the function of platelets in the early postoperative period and leads to increased bleeding after surgery and possibly increased use of blood products. In literature, we did not find any study examining the effect of diclofenac on platelet aggregation following cardiac surgery.

## METHODS

A prospective, cohort study was performed on patients admitted to the surgical ICU of the University Medical Centre Maribor (Slovenia), between May 2016 and December 2018. The study was approved by the Slovenian National Medical Ethics Committee on August 10, 2016 (Ref: 0120-430/2016-2). The study registration is ISRCTN14974395 (http://doi.org/10.1186/ISRCTN14974395).

In the study, adult patients undergoing elective cardiac surgery for CABG using CPB were included. Patients with a history of peptic ulcer, gastrointestinal bleeding, renal and liver insufficiencies, and allergy to nonsteroidal analgesics were excluded. Moreover, patients with increased bleeding during surgery, with massive blood transfusion, and hemodynamically unstable patients, who required a high dosage of vasoactive drugs, were excluded. Patients with prolonged CPB (duration > 200 minutes) and patients with increased bleeding after surgery, as defined by the chest tube drainage > 300 ml per hour during the first three hours after surgery, were also excluded.

Prior to operation, the following laboratory tests were performed: number of red blood cells, hemoglobin, hematocrit, and platelet count; platelet function (platelet aggregation to arachidonic acid, epinephrine, collagen, and adenosine diphosphate); hemostasis tests (prothrombin time, activated partial thromboplastin time, thrombin time, fibrinogen); urea and creatinine; and inflammation indicator (C-reactive protein [CRP]). During the operation, we recorded the type of surgery, the type and duration of anesthesia, the duration of CPB, the time of surgery, and the use of blood products (concentrated red blood cells, fresh frozen plasma, and platelet plasma).

After surgery, patients were randomized to one of two treatment groups using coin randomization. The diclofenac group received an intravenous infusion of 75 mg diclofenac in the form of 250 ml Neodolpasse^®^ (Fresenius Kabi) within 90 minutes, three hours after the cardiac surgery, and again after 12 hours. The control group received an infusion of 250 ml of saline (0.9% NaCl) within 90 minutes, three hours after surgery, and again after 12 hours. Both groups received analgesia with opioid analgesic piritramide (Dipidolor^®^), 0.05 mg/kg of body weight every six hours for postoperative pain relief. In addition, the patients were administered additional piritramide, 0.025 mg/ kg of body weight on request by means of nurse-controlled analgesia (NCA) to achieve adequate analgesia (faces rating scale and numerical rating scale [NRS] < 3)^[10]^. We evaluated and measured the intensity of pain every hour after the surgery. For this purpose, we used a faces rating scale for assessing pain in the intubated patient and NRS ranking in the awake patient. In pain assessment being 3 or more, the nurse applied an additional dose of piritramide.

We recorded the consumption of piritramide within 20 hours after surgery, the time until the tracheal tube was removed, and the time until discharge from the ICU. Immediately after the surgery and five hours and 20 hours after surgery, the following laboratory tests were performed: number of red blood cells, hemoglobin, hematocrit, platelet count, and platelet function (platelet aggregation to arachidonic acid, epinephrine, collagen, and adenosine diphosphate); hemostasis tests (prothrombin time, activated partial thromboplastin time, thrombin time, fibrinogen); urea and creatinine; inflammation indicator (CRP); and troponin and lactates.

Platelet aggregation was measured with four-channel platelet aggregometer (Hart Biologicals, United Kingdom). Both groups were followed up 20 hours after the surgery. Sternal drain blood loss was measured using the sternal drainage system Atrium at one-hour intervals until 20 hours after surgery. Time to extubation and duration of ICU stay were recorded. Unfavorable side effects were recorded during ICU stay.

### Statistical Analysis

Data were analyzed with the IBM SPSS Statistics software, version 25.0. Demographic data and baseline values were compared with Student’s *t*-test for independent samples and χ^2^, where appropriate. The analysis of variance (ANOVA) for repeated measurements was used to compare the laboratory tests between the treatment groups and the change over time. *P*<0.05 was considered statistically significant.

Sample size calculation was performed with the G Power 3.1.9.2. software. To detect a difference of 250 ml of blood loss and standard deviation (SD) of 300 ml with a probability level of 0.05 and a power of 0.90, a sample size of 64 patients was required. Sixty-six patients would detect a difference between the groups in opioid consumption of 6 mg and SD of 8 mg with a probability level of 0.05 and a power of 0.85. Expecting dropouts due to various reasons, including complications, we randomized 100 patients.

## RESULTS

**Fig. 1:**
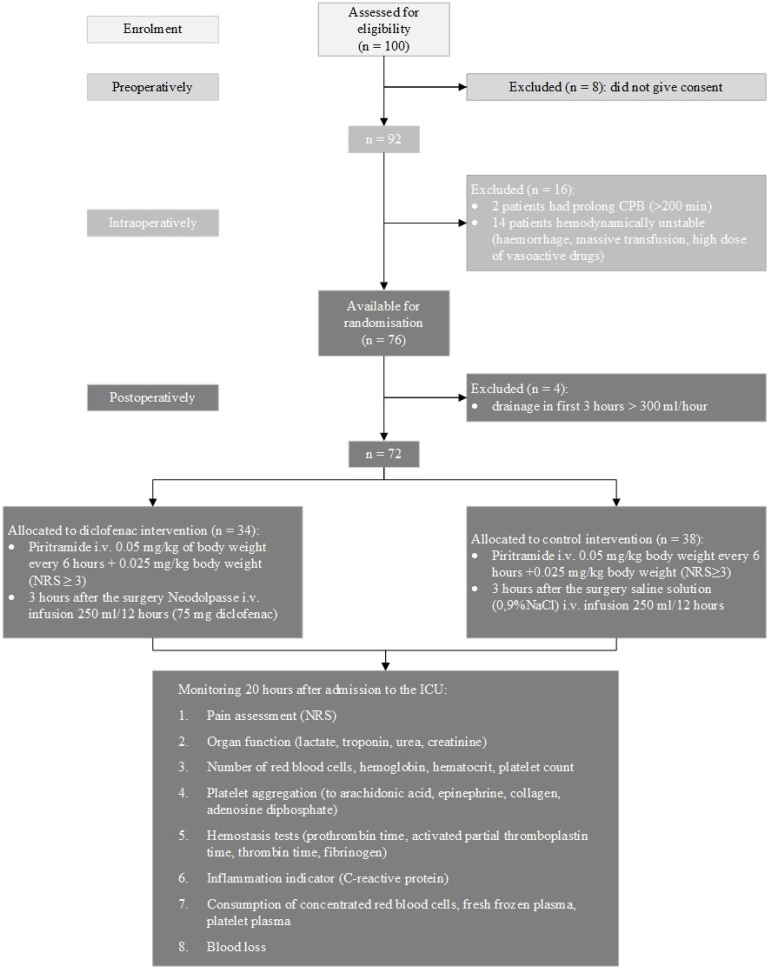
Study algorithm. CPB=cardiopulmonary bypass; ICU=intensive care unit; i.v.=intravenous; NRS=numerical rating scale

The Consolidated Standards of Reporting Trials (CONSORT) flow diagram of the study is shown in Figure 1. One hundred patients were screened for eligibility. Altogether, the data was completed for 72 patients who were included into the study.

The demographic data of patients, their medical history, the duration of CPB, the time of surgery, the time of anesthesia, the time to extubation, the time until discharge from the ICU, and the time to discharge from the hospital in both groups are shown in Table 1. There were no statistically significant differences in age, body weight, and height among the groups. The proportion of women in the diclofenac group was statistically significantly higher than in the control group. There were no statistically significant differences in the European System for Cardiac Operative Risk Evaluation (EuroSCORE II or ES II), the New York Heart Association (NYHA) rank, and the ejection fraction (EF) of left ventricle. There were statistically significant more patients with diabetes and hypercholesterolemia in the diclofenac group than in the control group. The incidence of other concomitant diseases did not significantly differ between the groups. There were also no significant differences between the groups regarding the type and duration of surgery and anesthesia and the time of CPB.

**Table 1:** Patients' basic characteristics, their classification in risk scores, accompanying diseases, and surgical features.

		Diclofenac (n =34)	Control (n = 38)
	Age (years)^a^	65±8	68±9
	Weight (kg)^a^	86±17	81±11
	Height (cm)^a^	172±8	171±8
	Sex (male/female)^b^	23/11*	34/4
	ES II^a^	2.43±2.5	3.0±4.1
NYHA rank^b^	NYHA 1	1	1
	NYHA 2	22	26
	NYHA 3	11	10
	NYHA 4	0	1
EF (%)^b^	Normal	25	27
	Moderate	8	10
	Severe	1	1
Concomitant diseases	Arterial hypertension^b^	30	29
	Diabetes^b^	15*	6
	Prior myocardial infarction^b^	13	15
	Hyperlipidemia^b^	11	16
	Hypercholesterolemia^b^	7*	1
	Asthma^b^	0	1
	COPD^b^	3	2
	Pulmonary hypertension^b^	2	5
	Atrial fibrillation^b^	2	6
Type of operation	Venous bypass^b^	32	34
	Arterial bypass^b^	29	33
	AVR^b^	5	11
	Time of CPB (min)^a^	101±22	112±27
	Duration of surgery (min)^a^	239±35	247±40
	Duration of anesthesia (min)^a^	291±34	298±41
	Type of anesthesia (F, S, SR)^b^	24:06:01	22:08:06
	Time to extubation (hours)^a^	9±5	10±6
	Nausea, vomiting^b^	2	1
	Discharge from ICU (hours)^a^	37±47	32±21
	Length of hospital stay (days)^a^	17±42	13±20
	Consumption of piritramide within 20 hours after surgery (mg)^a^	26±8	28±8

a mean values (± standard deviation)

b number of patients

* statistically significant difference (*P*<0.05) between groups (analysis of variance for repeated measurements)

AVR=aortic valve replacement; COPD=chronic obstructive pulmonary disease; CPB=cardiopulmonary bypass; EF=ejection fraction; ES II=European System for Cardiac Operative Risk Evaluation; F=Fentanyl; ICU=intensive care unit; NYHA=New York Heart Association; S=Sufentanil; SR=Sufentanil and Remifentanil

The time to extubation, the time of discharge from the ICU, and the length of hospital stay were not significantly different between the groups.

The consumption of piritramide did not differ significantly between the groups, but it was lower in the group that received diclofenac than in the control group. There were no statistically significant differences in the incidence of nausea and vomiting among the groups (Table 1).

Prothrombin time, activated partial thromboplastin time, and thrombin time were prolonged after the surgery and gradually returned to normal values within 20 hours, but there were no statistically significant differences between the groups (Table 2). Fibrinogen values decreased after surgery in both groups and there were no statistically significant differences between the groups.

**Table 2:** Blood, urine, and C-reactive protein (CRP) tests.

Group		Before the surgery	After the surgery	5 hours after surgery	20 hours after surgery
aPTT (sec)	Diclofenac	30.2±4.3	31.1±3.6	36.3±8.0	31.3±3.9†
	Control	31.6± 6.0	32.2±4.6	37.2±8.7	32.6±4.4†
PT (units)	Diclofenac	0.90±0.12	0.67±0.08	0.72±0.09	0.85±0.09†
	Control	0.89±0.11	0.65±0.09	0.70±0.07	0.82±0.09†
TT (sec)	Diclofenac	20.09±1.88	26.15±7.75*	49.87±37.53	21.03±3.37†
	Control	20.21±2.79	23.00±3.38	43.79±30.14	19.76±3.63†
Fibrinogen (g/l)	Diclofenac	4.41±1,33	2.94±0.80	3.05±0.88	3.87±0.82†
	Control	4.22±1,06	2.79±0.76	3.05±0.77	3.91±0.79†
Platelet count (10^9^/L)	Diclofenac	240±64	180±58	191±61	186±54†
	Control	237±114	169±92	182±85	182±78†
Platelet aggregation Adenosine diphosphate (%)	Diclofenac	73±16	75±18	73±19	76±18
	Control	70±20	78±22	79±20	80±11
Epinephrine (%)	Diclofenac	44±25	38±23	33±20	51±24†
	Control	43±29	36±26	42±32	61±27†
Arachidonic acid (%)	Diclofenac	27±32	34±31	29±34	35±31
	Control	29±34	35±33	42±35	49±32
Collagen (%)	Diclofenac	73±17	70±24	60±25	74±19
	Control	66±19	70±31	67±27	71±20
Urea (mmol/L)	Diclofenac	6.3±1.6	5.6±1.1		6.0±1.8
	Control	6.6±2.0	6.2±1.5		6.5±2.0
Creatinine (µmol/L)	Diclofenac	83±16	75±14		84±21
	Control	87±22	82±18		90±27
Lactate (mmol/L)	Diclofenac	1.9±0.7	2.8±1.7		1.9±0.5
	Control	2.3±0.9	3.6±2.0		2.2±0.8
Troponin (µmol/L)	Diclofenac		2.6±2.1	6.8±6.8	3.3±3.1
	Control		2.9±1.7	5.3±2.6	3.6±2.8
CRP (mg/L)	Diclofenac	6±4	4±2		33±15*
	Control	7±7	9±22		46±22

Mean values ± standard deviation

* statistically significant difference (*P*<0.05) between groups (analysis of variance for repeated measurements)

† statistically significant difference (*P*<0.05) within the group (analysis of variance for repeated measurements)

aPTT=activated partial thromboplastin time; PT=prothrombin time; TT=thrombin time

The platelet count decreased after the surgery in both groups, and 20 hours after surgery it was still lower than before the surgery. There were no statistically significant differences in the results of blood clotting tests, platelet count, and platelet aggregation tests between the groups.

Urea and creatinine did not differ significantly between the groups and did not significantly change after the operation. The lactate and troponin values increased significantly after the surgery in both groups showing no differences between them.

**Fig. 2:**
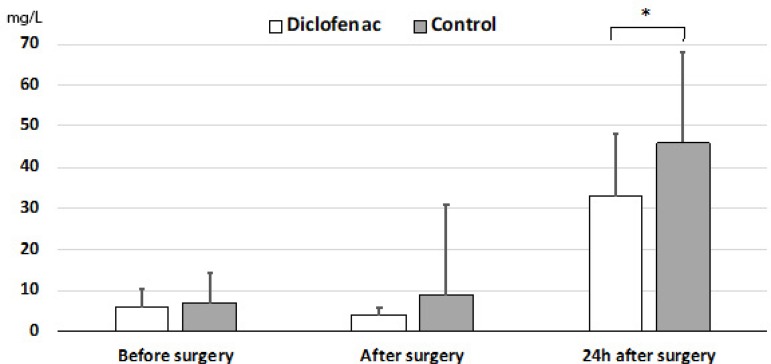
C-reactive protein levels before and after surgery.

CRP increased after surgery and 20 hours after surgery. CRP was statistically significantly lower in the diclofenac group than in the control group (Figure 2).

There were no statistically significant differences between groups in the consumption of blood transfusion and the use of fresh frozen plasma within 20 hours after surgery (Table 3). Within five hours and 20 hours after surgery, the amount of platelet transfusion was significantly higher in the control group than in the diclofenac group. There were no differences between the groups in the amount of blood loss within 20 hours after the surgery (Table 3).

**Table 3:** Consumption of blood and blood products during and after the surgery and blood loss after the surgery.

Group		Intraoperatively	1 hour after surgery	5 hours after surgery	20 hours after surgery
RBC (ml)	Diclofenac	180±247	82 ±174	121±296	121±296†
	Control	155±245	86±180	165±334	194±357†
FFP (ml)	Diclofenac	655±309	69 ±226	113±356	113±356
	Control	571±353	100±179	208±366	222±394
PP (ml)	Diclofenac	128±209	34±108*	33±107	33±107†
	Control	74±182	98±166	125±210	125±210†
Blood loss (ml)	Diclofenac		29±45	229±139	592±336
	Control		37±55	281±190	618±299

Mean values ± standard deviation

* statistically significant difference (*P*<0.05) between groups (analysis of variance for repeated measurements)

† statistically significant difference (*P*<0.05) within the group (analysis of variance for repeated measurements)

FFP=fresh frozen plasma; PP=platelet plasma; RBC=red blood cells

**Fig. 3:**
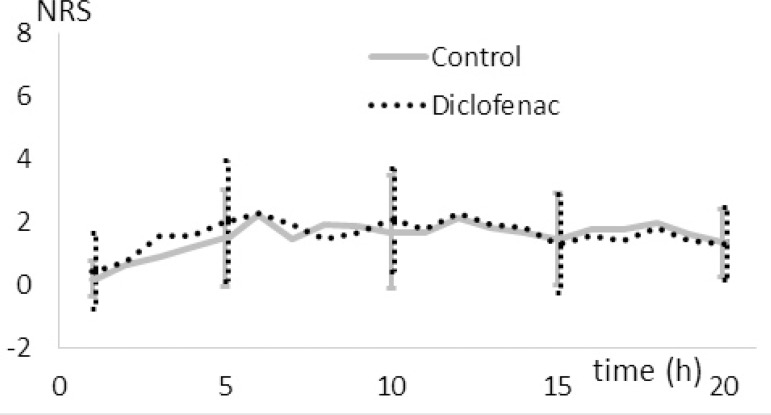
Intensity of pain after surgery. NRS=numerical rating scale

The intensity of pain measured with the NRS scale in combination with the faces rating scale (in the intubated patients) did not differ significantly between the groups within 20 hours after surgery (Figure 3).

Atrial fibrillation (AF) occurred in three patients (8%) of the diclofenac group and in seven patients (18%) of the control group. During hospitalization, confusion occurred in one patient (2%) and respiratory problems in three patients (8%) in both groups. One patient (2%) in the control group had pericardial effusion, bleeding after 20 hours, acute stroke, and larger pleural effusion; these side effects were not identified in the diclofenac group. There were no statistically significant differences in the incidence of postoperative adverse events between the groups.

## DISCUSSION

Our study evaluated the influence of diclofenac infusion on opioid consumption, coagulation (including platelet function), blood loss, and consumption of blood products after elective CABG surgery. The study did not confirm that the use of diclofenac would decrease the use of opioid analgesic piritramide following CABG surgery. A decrease in opioid consumption has been shown by some authors. In an older study, Fayaz et al.^[11]^ found statistically significantly lower consumption of opioid analgesic morphine in a group of patients receiving diclofenac and faster removal of the tracheal tube following CABG surgery. Hynninen et al.^[12]^ found statistically significantly lower morphine consumption in the group of patients receiving diclofenac compared to placebo. In the same study, morphine consumption in the group of patients receiving diclofenac was also decreased in comparison with the group receiving indomethacin and ketoprofen. Imantalab et al.^[13]^ compared the effects of morphine and diclofenac suppositories for the treatment of postoperative pain after CABG. They evaluated the visual analogue scale (VAS) at four-hour intervals after removing the tracheal tube for 24 hours. They found statistically significant reductions in pain in both groups, with no difference between the groups.

One of the possible reasons for the difference in our results could be our pain therapy protocol based on daily clinical practice used in most institutions, using the NCA approach rather than the patient-controlled analgesia (PCA) approach. Bainbridge et al.^[14]^ compared the intensity of pain after cardiac surgery using the NCA or PCA approach. They found lower pain intensity in patients with PCA *vs*. NCA. There were no significant differences between the groups regarding the ICU stay, the time of hospitalization, and the incidence of nausea and vomiting. The morphine consumption was higher in the PCA group. Similar results were obtained by Imantalab et al.^[15]^ in the study of 68 patients after a cardiac surgery using morphine for pain relieve with either the PCA or NCA approach. They demonstrated a higher use of morphine in the PCA approach in comparison to the NCA approach after heart surgery^[15]^. Four postoperative days after CABG, Kulik et al.^[16]^ used NSAIDs-naproxen and found reduced intensity of pain and slightly greater sternal drain blood loss, but the need for blood replacement was not greater. All the abovementioned reasons could explain the differences in our results. However, we must emphasize that our study had enough statistical power to detect a difference in piritramide consumption of 25% between the study groups. For this reason, the value of diclofenac in reducing opioid consumption in clinical setting after CABG surgery should be questioned.

For a more accurate assessment of postoperative pain, a strict protocol and additional patient education on pain relief would be required. Cogan et al.^[17]^ found that additional information in the brochure on pain relief received by patients after cardiac surgery did not reduce pain scores. On the other hand, in a study by Ziehm et al.^[18]^, the patients who were psychologically better prepared and receiving reassurance that they would not have major pain after the operation showed reduced pain scores after open-heart surgery. In our study using the NCA approach, we achieved a satisfactory pain relieve as shown by the low NRS scores on Figure 3. Detailed information about pain relief was provided to all the patients during an interview with the researcher one day before the surgery.

We expected that diclofenac would cause a disorder in the function of platelets, which would be minor and transient. However, there was no statistically significant difference in the use of diclofenac in platelet aggregation tests between the study groups. The platelet function decreased after heart surgery using CPB in both groups and slowly increased 20 hours after surgery. Clotting tests (prothrombin time, activated prothrombin time, thrombin time) were prolonged after surgery as a result of the effect of CPB on blood clotting in both groups of patients, with no differences between the groups. Twenty hours after surgery, clotting tests returned to baseline values as a result of normalization of hemostasis. Fibrinogen decreased after surgery in both groups, and after 20 hours, it approached preoperative values.

Finally, blood loss in the diclofenac group was not statistically significantly higher and there were no differences in the use of blood products.

In our study, urea and creatinine did not change significantly following the heart surgery. In the diclofenac group, they were comparable to the control group. We excluded patients with preoperative renal impairment. In a meta-analysis involving 20 controlled randomized trials, Acharya et al.^[19]^ monitored the renal function in patients who received NSAIDs after heart surgery. The incidence of renal impairment was not increased in the group receiving NSAIDs compared with the control group. The study included patients with a normal preoperative renal function^[19]^.

In our study, the time to extubation, the time of ICU stay, and the discharge from the hospital did not significantly differ between the groups.

Side effects of opioids, such as postoperative nausea and vomiting, were not common in our patients. Nausea and vomiting were reported by two patients in the diclofenac group, and one in the control group, with no significant differences between the groups.

Troponin and lactate increased after the surgery and decreased 20 hours after the surgery with no differences between groups.

CRP was statistically significantly lower in the diclofenac group after 20 hours, which could indicate the anti-inflammatory effects of NSAIDs. In some older studies, the NSAIDs have been shown to prevent postpericardiotomy syndrome^[20]^ and AF^[21]^ after cardiac surgery using CPB.

In a pooled data analysis of two multicentre, randomized studies, De Souza et al. did not identify an increased short-term risk of myocardial infarction, stroke, or increased mortality 30 days after cardiac surgery with the use of NSAIDs^[22]^. Howard et al.^[23]^ studied 178 patients treated for CABG. They compared the incidence of mortality, myocardial infarction, and bleeding among patients who received continuous infusion of 90 mg ketorolac and in the control group within 24 hours. In patients who received a 24-hour postoperative continuous infusion of ketorolac, no increased incidence of mortality, myocardial infarction, or significant bleeding was observed compared with the control group^[23]^. Qazi et al.^[24]^ found that patients who had been treated with ibuprofen after surgery for seven days had no greater risk of complications, such as myocardial infarction, hemorrhage, impaired renal function, and overall mortality than those treated with oxycontin.

Opinions on the use of NSAIDs in patients after CABG are still contradictory and require further research^[25]^. There are discussions about which NSAIDs are safer to use after a CABG based on thrombotic effects.

## CONCLUSION

In our study, the use of diclofenac did not reduce the use of NCA opiates after a cardiac surgery. Previous studies have not examined the effect of diclofenac on platelet aggregation following a cardiac surgery. We did not find any differences in platelet aggregation and other blood clotting tests in the group that received diclofenac. The diclofenac group also did not have increased bleeding and increased consumption of blood products in the early postoperative period. Lower CRP in the diclofenac group may indicate a reduced inflammatory response after CPB.

Our study showed that the use of diclofenac is safe after cardiac surgery; however, its value in reducing opioid consumption should be questioned.

**Table:** 

Authors' roles & responsibilities	
IO	Substantial contributions to the conception or design of the work; or the acquisition, analysis, or interpretation of data for the work; drafting the work or revising it critically for important intellectual content; final approval of the version to be published; agreement to be accountable for all aspects of the work in ensuring that questions related to the accuracy or integrity of any part of the work are appropriately investigated and resolved
MK	Drafting the work or revising it critically for important intellectual content; final approval of the version to be published
